# Historical and ecological drivers of the spatial pattern of Chondrichthyes species richness in the Mediterranean Sea

**DOI:** 10.1371/journal.pone.0175699

**Published:** 2017-04-13

**Authors:** María José Meléndez, José Carlos Báez, José Miguel Serna-Quintero, Juan Antonio Camiñas, Ignacio de Loyola Fernández, Raimundo Real, David Macías

**Affiliations:** 1 Centro Oceanográfico de Málaga, Instituto Español de Oceanografía (IEO), Fuengirola, Spain; 2 Departamento de Biología Animal, Universidad de Málaga, Málaga, Spain; 3 Centro Oceanográfico de Canarias, Instituto Español de Oceanografía (IEO), Santa Cruz de Tenerife, Spain; 4 Facultad de Ciencias de la Salud, Universidad Autónoma de Chile, Santiago de Chile, Chile; Aristotle University of Thessaloniki, GREECE

## Abstract

Chondrichthyes, which include Elasmobranchii (sharks and batoids) and Holocephali (chimaeras), are a relatively small group in the Mediterranean Sea (89 species) playing a key role in the ecosystems where they are found. At present, many species of this group are threatened as a result of anthropogenic effects, including fishing activity. Knowledge of the spatial distribution of these species is of great importance to understand their ecological role and for the efficient management of their populations, particularly if affected by fisheries. This study aims to analyze the spatial patterns of the distribution of Chondrichthyes species richness in the Mediterranean Sea. Information provided by the studied countries was used to model geographical and ecological variables affecting the Chondrichthyes species richness. The species were distributed in 16 Operational Geographical Units (OGUs), derived from the Geographical Sub-Areas (GSA) adopted by the General Fisheries Commission of the Mediterranean Sea (GFCM). Regression analyses with the species richness as a target variable were adjusted with a set of environmental and geographical variables, being the model that links richness of Chondrichthyes species with distance to the Strait of Gibraltar and number of taxonomic families of bony fishes the one that best explains it. This suggests that both historical and ecological factors affect the current distribution of Chondrichthyes within the Mediterranean Sea.

## Introduction

Species diversity gradients have been identified since the late 19^th^ century [1] affecting marine species richness [2]. Explaining the spatial trends of species distribution is of major importance in any biogeographic study, as they affect a set of species in a similar way. Marine species richness increases from the poles to the equator (latitudinal diversity gradient), similarly to what occurs in the terrestrial environment [2]. The climatic stability of the tropical seas has been proposed as the main mechanism explaining this pattern of species diversity [2]. Longitudinal distribution gradients in species richness in the marine environment have also been observed, and they are usually linked to historical processes [2]. Although the existence of these diversity patterns has been widely accepted, there are currently many ecosystems and specific environments for which the existence of a geographic trend in species richness is unknown [2]. Extinction-recolonization processes associated to geographical features have been used to explain the gradients observed in peninsulas (piece of land that is bordered by water but connected to mainland through one isthmus) [3–5], although these kinds of processes have never been linked before to explain analogous marine spatial patterns of species richness.

The Mediterranean Sea is a semi-closed sea, where oceanographic phenomena occur on a relatively small scale [6]. This sea could be seen as a marine “peninsula” as it was bordered by land, and only connected to the Atlantic Ocean through the Strait of Gibraltar, until the construction of the artificial Suez Canal. Moreover, during the Messinian salinity crisis [7–8] it suffered a process of extinction followed by a later recolonization. Thus, during this period, the Mediterranean Sea became a concentration basin, i.e., the contributions of rivers did not balance the evaporation losses [6]. Therefore, once interrupted the connection with the Atlantic Ocean, a process of gradual and almost complete desiccation of the Mediterranean Sea occurred in less than a thousand years. Two hundred thousand years later, the Atlantic water flow refilled the Mediterranean basin, in what is called the Zanclean flood.

Chondrichthyes, which include Elasmobranchii (sharks, rays, skates and sawfish) and Holoephali (chimaeras), have been successful in diverse ecosystems for over 400 million years. Despite their success, they are currently under threat as a result of human activities, including fishery [9]. Chondrichthyes play a key role in the ecosystems where they are found [10], many of them as apical predators. Some Elasmobranchii species are facing population declines in their distribution area [11–12]. Therefore, it is important to improve the knowledge of their spatial patterns and distribution areas. Recent studies have found that Elasmobranchii show a longitudinal gradient in the Mediterranean Sea [13]. However, no more than five batoid species could be considered Mediterranean endemic species [14]: the Maltese skate (*Leucoraja melitensis*), the speckled skate (*Raja polystigma*), the Mediterranean starry ray (*Raja asterias*), the rough ray (*Raja radula*), and the giant devilray (*Mobula mobular*).

The Suez Canal is an artificial sea-level waterway in Egypt, connecting the Mediterranean Sea to the Red Sea through the Isthmus of Suez. It was constructed by Ferdinand de Lesseps towards 1869. Since then, many alien species, considered as Lessepsian invasive species, have become established in the Mediterranean Sea making use of this pathway. Four Chondrichthyes are considered Lessepsian species [15–16]: *Carcharhinus altimus*, *Carcharhinus melanopterus*, *Himantura uarnak* and *Torpedo sinuspersici*, although the status of *Carcharhinus altimus* as Lessepsian species has been questioned [17]. On the other hand, it is possible to consider as vagrant or visitor species in the Mediterranean Sea the following: *Carcharhinus falciformis* [17], *Pristis pristis* [18], *Pristis pectinata* [18] and *Hydrolagus mirabilis* [19].

The objective of the present study was to analyze the spatial patterns of the distribution of Chondrichthyes species in the Mediterranean Sea, and testing historical and ecological explanatory hypothesis of these patterns by using spatial distribution models.

## Material and methods

### Data source

**Fig 1:**
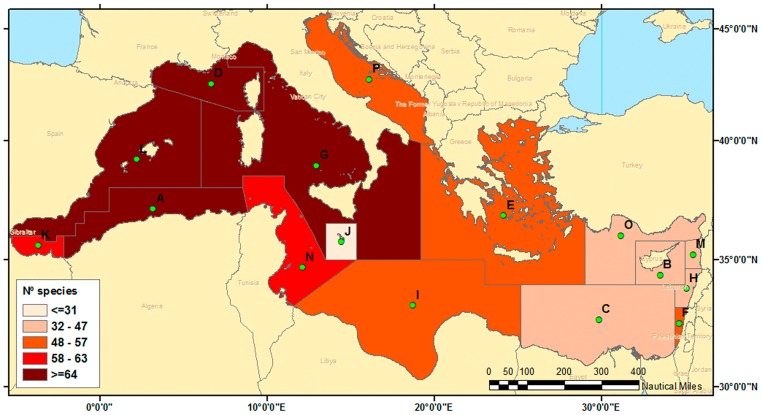
Geographic distribution of the Operational Geographical Units (OGUs) considered in this study. Green dots are the centroids of each OGU and the names of the countries designate the OGUs names.

The distribution of Chondrichthyes species recorded in local checklists is mainly referred to countries. For this reason, it is difficult to assign a species record to a point or local area. This issue has been solved by the General Fisheries Commission for the Mediterranean (GFCM, FAO) by adapting Res. GFCM/33/2009/2 (Establishment of Geographical Sub-Areas in the GFCM area). Geographical Sub-Areas (GSAs) have been defined according to multiple criteria, including the jurisdiction of each riparian country and distribution of fleets and fishing areas. GSAs were used in this study to facilitate the preparation of data. Likewise, the GFCM provides the Geographical Information System shapefiles. Nevertheless, the 30 Mediterranean GSAs defined by the GFCM (http://www.fao.org/gfcm/data/map-geographical-subareas/en/) were assembled into 16 Operational Geographical Units (OGUs) [19] by linking different GSAs of the same country, since most databases provide information about species presence in each country. The 16 OGUs, i.e., marine geographical areas, were built for operative reasons (see Fig 1 and Table 1).

**Table 1:** Countries and Geographical Sub-Areas (GSAs) corresponding to each Operational Geographical Unit (OGUs).

OGUs	GSAs	Countries
A	GSA 04	Algeria
B	GSA 25	Cyprus
C	GSA 26	Egypt
D	GSA07, GSA08	Francia and Monaco
E	GSA 20, GSA22, GSA 23	Greece
F	GSA27	Israel and Palestinian Territory
G	GSA09, GSA11,1, GSA11,2, GSA10, GSA16, GSA19	South and West Italy
H	GSA27	Lebanon
I	GSA21	Libya
J	GSA15	Malta
K	GSA3	Morocco
L	GSA01, GSA02, GSA05, GSA06	Spain
M	GSA27	Syrian Arab Republic
N	GSA12, GSA13,GSA14	Tunisia
O	GSA24	Turkey Meditarranean
P	GSA17, GSA18	Albania, Bosnia and Herzegovina, Croatia, Montenegro, Slovenia and East Italy

General sources (i.e., compendia of records) were used to perform the list of Chondrichthyes species cited in the areas included in each OGU [17, 20–23]. Likewise, data from the distribution maps produced by the IUCN Shark Specialist Group (available on the website www.redlist.org) until October 2015 were assembled. Furthermore, an active search of updated citations from the last 35 years was performed for each record considered as dubious, and other new records (Table 2) (S1 Table). According to the synonyms, accepted names from Fishbase were used [21].

**Table 2:** Chondrichthyes species recorded in the Mediterranean Sea, their occurrence at each Operational Geographical Units (OGUs) and IUCN status in a regional assessment. Key: ~ Lessepsian invasive species; § Vagrant and visitors; * Highly migratory species; & it presence is doubtful. CR, Critically Endangered; EN, Endangered; VU, Vulnerable; NT, Near Threatened; LC, Least Concern; NA, Not Assessed; DD, Data Deficient [25].

**Family**	**Genus + Species**	**Authority**	**Vernaculer Name**	**IUCN Region Global**	**IUCN Region Euro**	**IUCN Region MED**	**UGOs ocurrence**	**% of ocurrence**
Carcharhinidae	*Carcharhinus altimus* ~ §	(Springer, 1950)	Bignose shark	DD	DD	DD	A, B, C, F, H, I, K, L, M, O	62.5
Carcharhinidae	*Carcharhinus brachyurus*	(Günther, 1870)	Bronze whaler shark	NT	DD	DD	A, B, C, D, E, F, G, H, I, J, K, L, M, N, O,P	100
Carcharhinidae	*Carcharhinus brevipinna*	(Müller & Henle, 1839)	Spinner shark	NT	NA	NA	A, B, C, D, E, F, G, H, I, J, K, L, M, N, O,P	100
Carcharhinidae	Carcharhinus falciformis * §	(Müller & Henle, 1839)	Silky shark	NT	DD	NA	A, K, L, N	25
Carcharhinidae	*Carcharhinus limbatus*	(Müller & Henle, 1839)	Blacktip shark	NT	DD	DD	A, B, C, D, E, F, G, H, I, J, K, L, M, N, O,P	100
Carcharhinidae	*Carcharhinus melanopterus* ~	(Quoy & Gaimard, 1824)	Blacktip reef shark	NT	NA	NA	C, F, G, J, N, O	37.5
Carcharhinidae	*Carcharhinus obscurus*	(Lesueur, 1818)	Dusky shark	VU	DD	DD	A, D, F, G, I, J, K, L, M, N	62.5
Carcharhinidae	*Carcharhinus plumbeus*	(Nardo, 1827)	Sandbar shark	VU	EN	EN	A, B, C, D, E, F, G, H, I, J, K, L, M, N, O,P	100
Carcharhinidae	*Galeocerdo cuvier* §	(Péron & Lesueur, 1822)	Tiger shark	NT	DD	NA	G, L	12.5
Carcharhinidae	*Prionace glauca*	(Linnaeus, 1758)	Blue shark	NT	NT	CR	A, B, C, D, E, F, G, H, I, J, K, L, N, O,P	93.75
Carcharhinidae	*Rhizoprionodon acutus* § &	(Rüppell, 1837)	Milk shark	LC	NA	NA	E, G	12.5
Scyliorhinidae	*Galeus atlanticus*	(Vaillant, 1888)	Atlantic catshark	NT	NT	NT	A, K, L	18.75
Scyliorhinidae	*Galeus melastomus*	Rafinesque, 1810	Blackmouth catshark	LC	LC	LC	A, B, C, D, E, F, G, H, I, J, K, L, M, N, O,P	100
Scyliorhinidae	*Scyliorhinus canicula*	(Linnaeus, 1758)	Smallspotted catshark	LC	LC	LC	A, B, C, D, E, F, G, H, I, J, K, L, M, N, O,P	100
**Family**	**Genus + Species**	**Authority**	**Vernaculer Name**	**IUCN Region Global**	**IUCN Region Euro**	**IUCN Region MED**	**UGOs ocurrence**	**% of ocurrence**
Scyliorhinidae	*Scyliorhinus stellaris*	(Linnaeus, 1758)	Nursehound	NT	NT	NT	A, B, C, D, E, F, G, H, I, J, K, L, M, N, O,P	100
Sphyrnidae	Sphyrna lewini * §	(Griffith & Smith, 1834)	Scalloped hammerhead	EN	DD	NA	A, D, G, K, L	31.25
Sphyrnidae	Sphyrna mokarran * §	(Rüppell, 1837)	Great hammerhead	EN	DD	NA	A, B, C, D, E, G, I K, L, M, N	68.75
Sphyrnidae	*Sphyrna tudes*	(Valenciennes, 1822)	Smalleye hammerhead	VU	NA	NA	E, G, J, L, O, P	37.5
Sphyrnidae	Sphyrna zygaena *	(Linnaeus, 1758)	Smooth hammerhead	VU	DD	CR	A, B, C, D, E, F, G, H, I, J, K, L, M, N, O,P	100
Triakidae	*Galeorhinus galeus*	(Linnaeus, 1758)	Tope shark	VU	VU	VU	A, B, C, D, E, F, G, H, I, J, K, L, M, N, O,P	100
Triakidae	*Mustelus asterias*	Cloquet, 1819	Starry smoothhound	LC	NT	VU	A, B, C, D, E, F, G, H, I, J, K, L, M, N, O,P	100
Triakidae	*Mustelus mustelus*	(Linnaeus, 1758)	Smoothhound	VU	VU	VU	A, B, C, D, E, F, G, H, I, J, K, L, M, N, O,P	100
Triakidae	*Mustelus punctulatus*	Risso, 1827	Blackspot smoothhound	DD	VU	VU	A, B, C, D, E, F, G, H, I, K, L, M, N, O,P	93.75
Hexanchidae	*Heptranchias perlo*	(Bonnaterre, 1788)	Sharpnose sevengill shark	NT	DD	DD	A, B, C, D, E, F, G, H, I, J, K, L, M, N, O,P	100
Hexanchidae	*Hexanchus griseus*	(Bonnaterre, 1788)	Bluntnose sixgill shark	NT	LC	LC	A, B, C, D, E, F, G, H, I, J, K, L, M, N, O,P	100
Hexanchidae	*Hexanchus nakamurai*	Teng, 1962	Bigeye sixgill shark	DD	DD	DD	A, D, E, G, K, L, N, P	50
Alopiidae	Alopias superciliosus *	Lowe, 1841	Bigeye thresher	VU	EN	EN	A, B, C, D, E, F, G, H, I, J, K, L, M, N, O,P	100
Alopiidae	Alopias vulpinus *	(Bonnaterre, 1788)	Thresher shark	VU	EN	EN	A, B, C, D, E, F, G, H, I, J, K, L, M, N, O,P	100
Cetorhinidae	Cetorhinus maximus *	(Gunnerus, 1765)	Basking shark	VU	EN	EN	A, B, C, D, E, F, G, H, I, J, K, L, M, N, O,P	100
Lamnidae	Carcharodon carcharias *	(Linnaeus, 1758)	Great white shark	VU	CR	CR	A, B, C, D, E, F, G, H, I, J, K, L, M, N, O,P	100
Lamnidae	*Isurus oxyrinchus*	Rafinesque, 1810	Shortfin mako	VU	DD	CR	A, B, C, D, E, F, G, H, I, J, K, L, M, N, O,P	100
Lamnidae	Isurus paucus *	Guitart, 1966	Longfin mako	VU	DD	DD	A, K, L	18.75
**Family**	**Genus + Species**	**Authority**	**Vernaculer Name**	**IUCN Region Global**	**IUCN Region Euro**	**IUCN Region MED**	**UGOs ocurrence**	**% of ocurrence**
Lamnidae	Lamna nasus *	(Bonnaterre, 1788)	Porbeagle shark	VU	CR	CR	A, B, C, D, E, F, G, H, I, J, K, L, M, N, O,P	100
Odontaspididae	*Carcharias taurus*	Rafinesque, 1810	Sand tiger shark	VU	CR	CR	A, B, D, E, G, H, I, J, K, L, M, N, O,P	87.5
Odontaspididae	*Odontaspis ferox*	(Risso, 1810)	Smalltooth sand tiger	VU	CR	CR	A, B, C, D, E, F, G, H, I, J, K, L, M, N, O,P	100
Centrophoridae	*Centrophorus granulosus*	(Bloch & Schneider, 1801)	Gulper shark	NA	NA	NA	A, B, C, D, E, F, G, H, I, J, K, L, M, N, O,P	100
Centrophoridae	*Centrophorus uyato &*	Rafinesque, 1810	Little gulper shark	DD	VU	NA	A, B, D, E, F, G, H, I, J, K, L, M, N, O,P	93.75
Dalatiidae	*Dalatias licha*	(Bonnaterre, 1788)	Kitefin shark	NT	EN	VU	A, D, E, G, I, J, K, L, N, O, P	68.75
Echinorhinidae	*Echinorhinus brucus*	(Bonnaterre, 1788)	Bramble shark	DD	EN	EN	A, B, C, D, E, G, I, J, K, L, N, O, P	81.25
Etmopteridae	*Etmopterus spinax*	(Linnaeus, 1758)	Velvet belly	LC	NT	LC	A, B, D, E, F, G, I, J, K, L, N, O, P	81.25
Oxynotidae	*Oxynotus centrina*	(Linnaeus, 1758)	Angular roughshark	VU	VU	CR	A, B, C, D, E, F, G, H, I, J, K, L, M, N, O,P	100
Somniosidae	*Centroscymnus coelolepis*	Barbosa du Bocage & de Brito Capello, 1864	Portuguese dogfish	NT	EN	LC	A, D, G, I, K, L, N	43.75
Somniosidae	*Somniosus rostratus*	(Risso, 1827)	Little sleeper shark	DD	DD	DD	A, D, F, G, I, J, K, L, N	56.25
Squalidae	*Squalus acanthias*	Linnaeus, 1758	Spiny dogfish	VU	EN	EN	A, B, C, D, E, F, G, H, I, J, K, L, M, N, O,P	100
Squalidae	*Squalus blainville*	(Risso, 1827)	Longnose spurdog	DD	DD	DD	A, B, C, D, E, F, G, H, I, J, K, L, M, N, O,P	100
Squalidae	*Squalus megalops*	(MacLeay, 1881)	Shortnose spurdog	DD	DD	DD	A, D, G, K, L, N	37.5
Squatinidae	*Squatina aculeata*	Cuvier, 1829	Sawback angelshark	CR	CR	CR	A, C, D, E, G, I, K, L, N, O	62.5
Squatinidae	*Squatina oculata*	Bonaparte, 1840	Smoothback angelshark	CR	CR	CR	A, B, C, D, E, F, G, H, I, J, K, L, M, N, O,P	100
Squatinidae	*Squatina squatina*	(Linnaeus, 1758)	Angelshark	CR	NA	CR	A, B, C, D, E, F, G, H, I, J, K, L, M, N, O,P	100
**Family**	**Genus + Species**	**Authority**	**Vernaculer Name**	**IUCN Region Global**	**IUCN Region Euro**	**IUCN Region MED**	**UGOs ocurrence**	**% of ocurrence**
Dasyatidae	*Dasyatis centroura*	(Mitchill, 1815)	Roughtail stingray	LC	VU	VU	A, B, C, D, E, F, G, H, I, J, K, L, M, N, O,P	100
Dasyatidae	*Dasyatis chrysonota &*	(Smith, 1828)	Blue stingray	LC	NA	NA	F, N	12.5
Dasyatidae	*Dasyatis marmorata*	(Steindachner, 1892)	Marbled stingray	DD	DD	DD	F, K, N, O	25
Dasyatidae	*Dasyatis pastinaca*	(Linnaeus, 1758)	Common stingray	DD	VU	VU	A, B, C, D, E, F, G, H, I, J, K, L, M, N, O,P	100
Dasyatidae	*Dasyatis tortonesei*	Capapé, 1975	Tortonese´s stingray	DD	VU	VU	A, B, C, D, E, F, G, H, I, K, L, M, N, O	87.5
Dasyatidae	*Himantura uarnak* ~	(Forsskael, 1775)	Honeycomb whipray	VU	NA	NA	C, F, H, O	25
Dasyatidae	Pteroplatytrygon violacea *	(Bonaparte, 1832)	Pelagic stingray	LC	LC	LC	A, C, D, E, F, G, I, J, K, L, N, O,P	81.25
Dasyatidae	*Taeniura grabata*	(Geoffroy Saint-Hilaire, 1817)	Round fantail stingray	DD	DD	DD	A, C, F, G, H, I, M, N,O	56.25
Gymnuridae	*Gymnura altavela*	(Linnaeus, 1758)	Spiny butterfly ray	VU	CR	CR	A, B, C, D, E, F, G, H, I, J, K, L, M, N, O,P	100
Mobulidae	Mobula mobular *	(Bonnaterre, 1788)	Giant devilray	EN	NA	EN	A, B, C, D, E, F, G, H, I, J, K, L, M, N, O,P	100
Myliobatidae	*Myliobatis aquila*	(Linnaeus, 1758)	Common eagle ray	DD	VU	VU	A, B, C, D, E, F, G, H, I, J, K, L, M, N, O,P	100
Myliobatidae	*Pteromylaeus bovinus*	(Geoffroy Saint-Hilaire, 1817)	Bullray	DD	CR	CR	A, B, C, D, E, F, G, H, I, J, K, L, M, N, O,P	100
Rhinopteridae	*Rhinoptera marginata*	(Geoffroy Saint-Hilaire, 1817)	Lusitanian cownose ray	NT	DD	DD	A, B, C, D, E, F, G, H, I, J, K, L, M, N, O,P	100
Pristidae	*Pristis pectinata* §	Latham, 1794	Smalltooth sawfish	CR	CR	CR	D, E, F, G, H, K, L, M, P	56.25
Pristidae	*Pristis pristis* §	(Linnaeus, 1758)	Common sawfish	CR	CR	CR	D, E, G, L, P	31.25
Rajidae	*Dipturus batis*	(Linnaeus, 1758)	Common skate	CR	CR	CR	A, D, E, G, J, K, L, O, P	56.25
Rajidae	*Dipturus nidarosiensis*	(Storm, 1881)	Norwegian skate	NT	NA	NA	G	6.25
Rajidae	*Dipturus oxyrinchus*	(Linnaeus, 1758)	Sharpnose skate	NT	NA	NT	A, B, C, D, E, F, G, H, I, J, K, L, M, N, O,P	100
**Family**	**Genus + Species**	**Authority**	**Vernaculer Name**	**IUCN Region Global**	**IUCN Region Euro**	**IUCN Region MED**	**UGOs ocurrence**	**% of ocurrence**
Rajidae	*Leucoraja circularis*	(Couch, 1838)	Sandy skate	EN	NA	CR	A, C, D, E, G, I, K, L, N, O, P	68.75
Rajidae	*Leucoraja fullonica*	(Linnaeus, 1758)	Shagreen skate	VU	NA	CR	A, D, E, F, G, H, J, K, L, N, O, P	75
Rajidae	*Leucoraja melitensis*	Clark, 1926	Maltese skate	CR	NA	NA	A, D, G, I, J, N	37.5
Rajidae	*Leucoraja naevus*	(Müller & Henle, 1841)	Cuckoo skate	LC	NA	NT	A, B, D, E, F, G, H, I, J, K, L, N, O	81.25
Rajidae	*Raja africana*	Capepe, 1977	African skate	NA	NA	NA	N	6.25
Rajidae	*Raja asterias*	Delaroche, 1809	Atlantic starry skate	NT	NA	NT	A, B, C, D, E, F, G, H, I, J, K, L, M, N, O,P	100
Rajidae	*Raja brachyura*	Lafont, 1871	Blonde skate	NT	NT	NT	A, D, E, G, J, K, L, N	50
Rajidae	*Raja clavata*	Linnaeus, 1758	Thornback skate	NT	NT	NT	A, B, C, D, E, F, G, H, I, J, K, L, M, N, O,P	100
Rajidae	*Raja miraletus*	Linnaeus, 1758	Twineye skate	LC	LC	LC	A, B, C, D, E, F, G, H, I, J, K, L, M, N, O,P	100
Rajidae	*Raja montagui*	Fowler, 1910	Spotted skate	LC	LC	LC	A, C, D, E, G, H, I, K, L, N, O, P	75
Rajidae	*Raja polystigma*	Regan, 1923	Speckled skate	LC	NA	NA	A, D, E, G, I, K, L, N, O, P	62.5
Rajidae	*Raja rádula*	Delaroche, 1809	Rough skate	EN	NA	NA	A, B, C, D, E, F, G, H, I, J, L, M, N, O,P	93.75
Rajidae	*Raja undulata*	Lacepède, 1802	Undulate skate	EN	NT	NT	A, D, E, F, G, H, K, L, N, O, P	68.75
Rajidae	*Rostroraja alba*	(Lacepède, 1803)	White skate	EN	CR	EN	A, D, E, G, I, J, K, L, N, O, P	68.75
Rhinobatidae	*Rhinobatos cemiculus*	Geoffroy Saint-Hilaire, 1817	Blackchin guitarfish	EN	EN	EN	A, B, C, D, E, F, G, H, I, J, K, L, M, N, O,P	100
Rhinobatidae	*Rhinobatos rhinobatos*	(Linnaeus, 1758)	Common guitarfish	EN	EN	EN	A, B, C, D, E, F, G, H, I, J, K, L, M, N, O,P	100
Torpedinidae	*Torpedo marmorata*	Risso, 1810	Spotted torpedo ray	DD	LC	LC	A, B, C, D, E, F, G, H, I, J, K, L, M, N, O,P	100
**Family**	**Genus + Species**	**Authority**	**Vernaculer Name**	**IUCN Region Global**	**IUCN Region Euro**	**IUCN Region MED**	**UGOs ocurrence**	**% of ocurrence**
Torpedinidae	*Torpedo nobiliana*	Bonaparte, 1835	Great torpedo ray	DD	LC	LC	A, B, C, D, E, F, G, H, I, J, K, L, M, N, O,P	100
Torpedinidae	*Torpedo sinuspersici* ~	Olfers, 1831	Variable torpedo ray	DD	NA	NA	M	6.25
Torpedinidae	*Torpedo torpedo*	(Linnaeus, 1758)	Ocellate torpedo ray	DD	LC	LC	A, B, C, D, E, F, G, H, I, J, K, L, M, N, O,P	100
Chimaeridae	*Chimaera monstrosa*	Linnaeus, 1758	Rabbitfish	NT	NT	NT	A, B, D, E, G, H, J, K, L, M, N, O,P	81.25
Chimaeridae	Hydrolagus mirabilis §	(Collett, 1904)	Large-eyed rabbitfish	NT	LC	NA	M	6.25

On the other hand, some records, i.e., *Sphyrna tudes* and *Galeocerdo cuvier* have been questioned. However, the remains of *G*. *cuvier* (deposited in the Museum Alborania of Malaga) were examined and they certainly correspond to *G*. *cuvier*. Moreover, recently was confirmed the presence of *G*. *cuvier* from Mediterranean Sea by captures of juveniles specimens in Libya [24]. Therefore, in this study, these citations have been considered as records of vagrant species.

### Methods and statistical analysis

The Chondrichtyes Species Richness (CSR) of each OGU was obtained from the total number of species present by OGU (S1 Table). Latitude and longitude of each OGU were calculated with a geographic information system (ArcGis 10.3 program). Centroid coordinates of each OGU were calculated and their values of latitude and longitude were inferred. These values were used to assess the existence of a geographical gradient in CSR.

The correlation between the CSR of each OGU with the latitude (abbreviated as LAT) and longitude (abbreviated as LON) of each OGU centroid (to assess the spatial gradient) [26–27] was tested.

Linear multiple regressions were performed to test monotonic responses of CSR predicted by several historical, ecological and environmental factors. The best fit among significant regressions, with different degrees of freedom in accordance with the highest F-value, was selected. The normality of variables was previously tested with the Shapiro-Wilk test [28]. Six variables were selected: Sea Surface Temperature (SST), Salinity in Depth (SD), the OGU Area (OAR), number of taxonomic Families of bony fishes per OGU (FAM), Distance from the centroid of each OGU to the Strait of Gibraltar (DISTG) and Distance from the centroid of each OGU to the Suez Channel (DISTS) (Table 3).

**Table 3:** Independent variables used in this study for each Operational Geographical Unit (OGU). Key: LAT, latitude (degrees); LON, longitude (degrees); OAR, the OGU area (km^2^); DISTG, distance from the centroid of each OGU to the Strait of Gibraltar (km); DISTS, distance from the centroid of each OGU to the Suez Canal(km); SST, sea surface temperature (°C); SD, salinity in depth (PSU); FAM, number of taxonomic families of bony fishes for each OGU.

UGOs	LON	LAT	OAR	DISTG	DISTS	SST	SD	FAM
A	3.14	37.10	126138.67	712.62	2522.6	19.61	38.11	139
B	33.50	34.39	44932.56	3225.50	408.3	22.25	38.76	100
C	29.82	32.62	261633.71	2945.68	270.5	21.95	38.78	127
D	6.61	42.52	87910.26	1235.27	2518.4	17.62	38.41	134
E	24.10	36.83	407799.36	2441.00	970.6	19.93	38.84	141
F	34.62	32.48	7781.85	3341.64	248.1	23.10	38.91	156
G	12.91	38.94	538842.39	1548.34	1862.1	19.60	38.54	154
H	35.05	33.85	14336.88	3358.43	400.4	22.76	38.79	107
I	18.70	33.18	363869.95	2024.93	1153.9	21.31	38.68	101
J	14.40	35.74	26770.88	1636.75	1586.3	20.12	38.67	128
K	-3.76	35.59	22541.21	147.01	3036.8	18.77	38.33	135
L	2.15	39.19	252105.06	719.00	2680.3	19.13	38.41	145
M	35.45	35.22	10501.72	3381.54	561.4	22.61	38.78	117
N	11.44	34.77	128629.12	1391.91	1730.6	19.92	38.16	117
O	31.15	36.00	112061.71	3023.94	599.7	21.16	38.79	141
P	16.05	42.71	137504.35	1928.50	1918.7	18.30	38.55	58

The variables, i.e., OAR, DISTG and DISTS, were calculated with ArcGIS spatial analysis tools (ArcGIS 10.3 program), while SST and SD were estimated from the data provided by NOAA (National Oceanic and Atmospheric Administration) [29]. They were referred to the mean between the years 2005–2010 for the available data. The number of taxonomic families of bony fishes per OGU (FAM) was obtained from Fishbase [21]. In a second step, the Lessepsian, vagrant, visitors, and highly migratory species were removed from the analysis. According to the specialist group of sharks of the International Commission for the Conservation of Atlantic Tunas (ICCAT), the Elasmobranchii species considered as pelagic, oceanic and highly migratory in the ICCAT Convention area are: *Carcharodon carcharias*, *Isurus paucus*, *Lamna nasus*, *Cetorhinus maximus*, *Alopias superciliosus*, *A*. *vulpinus*, *Carcharhinus falciformis*, *Sphyrna lewini*, *S*. *mokarran*, *S*. *zygaena*, *Pteroplatytrygon violacea* and *Mobula mobular* [30].

## Results

A total of 89 species of Chondrichthyes were recorded in the Mediterranean Sea, of which 49 are sharks, 38 are rays and 2 Holocephali species (*Chimaera monstrosa* and *Hydrolagus mirabilis*). Annex (see S1 Table) shows the list of species by OGU, including their habitat (pelagic or demersal). The average number of Chondrichthyes observed species by OGU is 66. Of the Chondrichthyes species from the Mediterranean Sea, 57 species were assessed in the IUCN Red List of Threatened Species [25].

In the Mediterranean Sea, a total of 41 species of Chondrichthyes are found in all the 16 OGUs. Of these 41 species, 25 are sharks and 16 are Batoidea.

A significant negative correlation between CSR and the longitude of the centroid of each OGU (LON) (r = -0.822; P < 0.001) was found, but the highest correlation was observed with the distance to the Strait of Gibraltar (see Table 4). If Lessepsian, vagrant, visitors, and highly migratory species of Chondrichthyes previously mentioned are removed from the analysis, taken together and separately, similar results were obtained (Table 4).

**Table 4:** Pearson correlation coefficient between the independent variables used in this study for each Operational Geographical Unit (OGU), and dependent variables: Chondrichthyes species Richness (CSR), Chondrichthyes species Richness without Lessepsian invasive species (LCSR); Chondrichthyes species Richness without vagrant and visitors (VCSR); Chondrichthyes species Richness without highly migratory species (HCSR); Chondrichthyes species Richness without Lessepsian invasive species, highly migratory species, vagrant and visitors together (ALLCSR). Pearson correlation coefficient is shown (significance in brackets). Key: LAT, latitude (degrees); LON, longitude (degrees); OAR, the OGU area (km^2^); DISTG, distance from the centroid of each OGU to the Strait of Gibraltar (km); DISTS, distance from the centroid of each OGU to the Suez Canal (km); SST, sea surface temperature (°C); SD, salinity in depth (PSU); FAM, number of taxonomic families of bony fishes per OGU. ** Significant correlation.

	LON	LAT	OAR	DISTG	DISTS	SST	SD	FAM
CSR	-0.822** (<0.001)	0.489 (0.055)	0.423 (0.103)	-0.824** (<0.001)	0.804** (<0.001)	-0.736** (0.001)	-0.738** (0.001)	0.463 (0.071)
LCSR	-0.845** (<0.001)	0.539** (0.031)	0.430 (0.096)	-0.846** (<0.001)	0.833** (<0.001)	-0.781** (<0.001)	-0.752** (0.001)	0.406 (0.119)
VCSR	-0.804** (<0.001)	0.425 (0.1)	0.368 (0.161)	-0.809** (<0.001)	0.77** (<0.001)	-0.710** (0.002)	-0.744** (0.001)	0.443 (0.086)
HCSR	-0.773** (<0.001)	0.508** (0.045)	0.442 (0.087)	-0.774** (<0.001)	0.76** (<0.001)	-0.726** (0.001)	-0.690** (0.003)	0.452 (0.076)
ALLCSR	-0.82** (<0.001)	0.496 (0.051)	0.387 (0.139)	-0.823** (<0.001)	0.797** (<0.001)	-0.763** (0.001)	-0.758** (0.001)	0.370 (0.159)

The best model selected for the Chondrichthyes Species Richness (CSR) in the Mediterranean Sea, according to R^2^ and F values, was a multiple linear model between CSR, as a dependent variable, and DISTG and FAM as explanatory variables (R^2^ adjusted = 0.746; F = 22.996; P< 0.001). The model is shown as follows (order of variables in the model related to their weight according to β value):

CSR=65.296−0.006×DISTG+0.113×FAM

If Lessepsian, vagrant, visitors, and highly migratory species of Chondrichthyes previously mentioned are removed from the analysis, taken together and separately, then DISTG was the only or the most explanatory variable (Table 5). The number of species in the Eastern Mediterranean region might have increased recently due to Lessepsian migration of species of the group considered here. By discarding the Lepssepsian species, the linear model between species richness and dependent variables improved its fitting (R^2^adjusted for LCSR = 0.766 vs. R^2^adjusted for CSR = 0.746).

**Table 5:** Results of the multiple lineal regression models among Chondrichthyes Species Richness (CSR) excluding only Lessepsian (LCSR), only vagrant, visitors (VCSR), and only highly migratory species of Chondrichthyes previously mentioned (HCSR), and excluding all of them pooled together (ALLCSR) versus historical, ecological and environmental factors. Key: DISTG, distance from the centroid of each OGU to the Strait of Gibraltar (km); OAR, the OGU area (km^2^).

	R^2^adjusted	F-Fisher	P of the model	Variables in the model
LCSR	0.766	25.547	<0.001	DISTG; OAR
VCSR	0.631	26.661	<0.001	DISTG
HCSR	0.570	20.906	<0.001	DISTG
ALLCSR	0.654	29.322	<0.001	DISTG

## Discussion

According to our results, the best explanatory variables of CSR distribution are the distance from the centroid of each OGU to the Strait of Gibraltar (DISTG) and the number of taxonomic families of bony fishes in each OGU. Moreover, if Lessepsian, vagrant, visitors, and highly migratory species of Chondrichthyes previously mentioned are removed from the analysis, taken together and separately, then DISTG is the most important variable in all the cases.

Thus, two possible explanatory hypotheses could support this geographical distribution in CSR: *Historical causes* (i.e., extinction followed by a recolonization process and a “peninsula effect”), and *the Mediterranean Sea as an ecological sink*. Due to the Messinian salinity crisis aforementioned, the Mediterranean suffered almost total desiccation, becoming extinct many of the marine species; some of them recolonizing the Mediterranean Sea once opened and refilled again with Atlantic waters [7]. This could explain the low level of endemism of Chondrichthyes in the Mediterranean Sea [9,14]. Thus, our results suggest that this event has left a mark on the species richness distribution pattern. This pattern is consistent with the hypothesis of the extinction-recolonization process of the Mediterranean Sea by Chondrichthyes from the Atlantic Ocean. This process could persist at present by the “peninsula effect” caused by the role of the Strait of Gibraltar as an isthmus for the entrance of the species to the Mediterranean Sea, making the proximities of the Strait of Gibraltar more accessible to the Atlantic species. Consequently, the Messinian crisis and the geographical structure of the Mediterranean Sea as a marine peninsula, could be the main drivers of the longitudinal gradient of CSR reported before [13], and similarly to the gradient of species richness found in terrestrial peninsulas from the isthmus to the furthermost edge [3–5].

The other explanation for this longitudinal gradient could be related to the possible role of the Mediterranean Sea as an ecological sink. With regard to this hypothesis, when modeling CRS by removing from the analysis the highly migratory species from the Mediterranean Sea (this species set could be mainly affected by the ecological sink effect) a similar model was obtained (to see Table 4). Therefore, the basis of the ecological sink hypothesis does not explain properly the Mediterranean CSR.

With regard to the other ecological and environmental factors, distance from the centroid of each OGU to the Suez Canal (DISTS), latitude (LAT), salinity in depth (SD), and sea surface temperature (SST), the relation observed was in some or all cases significant. However, in all cases, CSR was best correlated with DISTG than with the other variables (to see Table 4). This suggests that the correlation between CSR and the other variables is based on the DISTG-CSR relationship.

Latitude is only correlated with CSR, without considering Lessepsian species (LCSR) and highly migratory species (HCSR). However, latitude is related to longitude, as the easternmost OGUs are also the southernmost ones and, consequently, the most outlying areas to the Strait of Gibraltar.

The second variable used in the multivariate model was the number of taxonomic families of bony fishes per OGU (FAM), showing a positive relation with CSR. FAM summarized the availability of different prey types at each OGU, suggesting that CSR is also affected by some ecological processes.

In conclusion, the extinction-recolonization process, the “peninsula effect”, and the availability of preys altogether, constitute the main historical and ecological factors that could explain the current distribution of Chondrichthyes species in the Mediterranean Sea.

## Supporting information

S1 Table Checklist of Chondrichthyes species recorded in the Mediterranean Sea, and occurrence for each Operational Geographical Units (OGUs) and reference notes. According to the synonyms, accepted names from Fishbase were used [21].
(XLSX)
